# The Sensitivity of Ear-EEG: Evaluating the Source-Sensor Relationship Using Forward Modeling

**DOI:** 10.1007/s10548-020-00793-2

**Published:** 2020-08-24

**Authors:** Arnd Meiser, Francois Tadel, Stefan Debener, Martin G. Bleichner

**Affiliations:** 1 Department of Psychology, University of Oldenburg, Oldenburg, Germany; 2 Montreal Neurological Institute, McGill University, Montreal, Canada

**Keywords:** Ear-EEG, Ear-centered sensing, Forward modeling, cEEGrid, Cortical folding, Sensitivity map

## Abstract

Ear-EEG allows to record brain activity in every-day life, for example to study natural behaviour or unhindered social interactions. Compared to conventional scalp-EEG, ear-EEG uses fewer electrodes and covers only a small part of the head. Consequently, ear-EEG will be less sensitive to some cortical sources. Here, we perform realistic electromagnetic simulations to compare cEEGrid ear-EEG with 128-channel cap-EEG. We compute the sensitivity of ear-EEG for different cortical sources, and quantify the expected signal loss of ear-EEG relative to cap-EEG. Our results show that ear-EEG is most sensitive to sources in the temporal cortex. Furthermore, we show how ear-EEG benefits from a multi-channel configuration (i.e. cEEGrid). The pipelines presented here can be adapted to any arrangement of electrodes and can therefore provide an estimate of sensitivity to cortical regions, thereby increasing the chance of successful experiments using ear-EEG.

**Electronic supplementary material:**

The online version of this article (10.1007/s10548-020-00793-2) contains supplementary material, which is available to authorized users.

## Introduction

Ear-EEG (electroencephalography) opens up new possibilities to record brain activity beyond the lab with minimal inconvenience for a person (Debener et al. (2015); Bleichner and Debener (2017)). For instance, ear-EEG could become an integral part for medical applications like epilepsy- (Zibrandtsen et al. (2017)) or sleep-monitoring (Looney et al. (2016); Nakamura et al. (2017)) as well as attention tracking (Mirkovic et al. (2016)) or as part of hearing devices (Fiedler et al. (2016); Denk et al. (2018)). The goal of ear-EEG is to measure brain-electrical activity in natural, daily life conditions and over long periods of time. With classical, stationary EEG, including a larger number of scalp electrodes, cables and additional necessary equipment, measuring under real-life conditions is difficult. To this end, an ear-EEG system ideally avoids those issues and is no more visible or distracting than a hearing device or glasses. Of course, this puts constraints on the number and the placement of the few available electrodes.

There are two different approaches tackling this problem: one is in-ear-EEG, where electrodes are either placed in the outer ear canal or the concha (Kidmose et al. (2012); Looney et al. (2012); Lee et al. (2014); Fiedler et al. (2017)). The second approach is around-the-ear EEG (Debener et al. (2015); Bleichner and Debener (2017)), placing electrodes closely to the ear in a circular arrangement around the outer ear (Debener et al. (2015); Bleichner and Debener (2017)). In both cases, the number of electrodes is limited and their position is constrained to a relatively small area in or around the ears. While in-ear-EEG is the less visible solution, around-the-ear-EEG has the advantage of larger inter-electrode distances, which in turn allows for the recording of larger amplitude signals. These two approaches stress the balance researchers have to find in the development and application of ear-EEG. On the one hand, the aim is to acquire data as conveniently and unobtrusively as possible. On the other hand, it is crucial to collect high-quality signals, in order to compensate for the loss of spatial information. The overarching goal is to capture cortical signals as reliably as possible for extended periods of time and in a way users can accept.

With ear-EEG, event-related potential (ERP) components like the P300 (Looney et al. (2012); Debener et al. (2015)), the N1 (Debener et al. (2015); Bleichner et al. (2016); Mirkovic et al. (2016)) as well as oscillations in the alpha frequency range (Looney et al. (2011); Debener et al. (2015); Mikkelsen et al. (2015)) can be reliably recorded. Ear-EEG is of special interest for beyond-the-lab studies of auditory attention or speech intelligibility in noisy environments (Denk et al. (2017, 2018); Nogueira et al. (2019)). While these studies may be regarded as demonstrating a proof of concept technology readiness level, most effects were found to be smaller than those found with classical EEG. Since EEG acquisition in ear-EEG is reduced to locations in- and around the ear, ear-EEG cannot be expected to reach the sensitivity of high-density cap-EEG for capturing brain-electrical activity. To date, it is not well understood how ear-EEG electrodes should be optimally arranged, and where they should be located, in order to minimize this loss of information compared to cap EEG.

EEG source localization and related forward models can help in finding the optimal electrode configuration for a particular application. Forward models explain how an electric field propagates from a specific source through the head volume (grey matter, cerebrospinal fluid (CSF), skull and skin) to scalp EEG electrodes. To simulate the propagation of an electric field through the head, there must be a representation of its source. Current dipoles are well suited for representing EEG source signals, which originate from large-scale synchronization of post-synaptic potentials in pyramidal cortical neurons arranged in parallel to each other and thereby generating open electrical fields (Hallez et al. (2007)). In a forward model, sources of cortical activity are therefore assumed to be one or more active equivalent current dipoles with a certain orientation and amplitude. To place sources and to estimate their propagation through the head volume in a realistic way, computing an EEG forward model requires detailed information about the geometry and conductivity of the different tissues. In a pioneering study, Kappel et al. (2019) extended a classical anatomical model by adding the ear canal to the head model, thereby achieving more realistic conductivity estimates for in-ear-EEG. To test the sensitivity of different electrode configurations for the signals arising from single dipole sources, they used forward modeling based on an individual head model segmenting the head volume into scalp, outer skull, inner skull and brain. Their study makes an important step by modeling the individual head in order to receive more realistic recordings of ear-EEG and apply forward modeling to around-the-ear-EEG. In addition to using a realistic head model, we explore the role of source depth, position and orientation for ear-EEG signals.

In the localization of cortical activity from classical EEG recordings, the influence of orientation, position and depth of a source on localization accuracy has been the subject of several studies (Roth et al. (1993); Yvert et al. (1996); Whittingstall et al. (2003)). For an extension to ear-EEG, we use the cEEGrid ear-EEG (Debener et al. (2015); Bleichner et al. (2016)) to quantify how sensitive ear-EEG is to the orientation, position and depth of active cortical tissue. Since the cortical structure of a person is highly individual (Kanai and Rees (2011); Llera et al. (2019)), large inter-individual differences in the resulting ear-EEG signals can be expected, due to individual differences in cortical folding. Therefore, we first demonstrate the complex sensor-source relationship on the example of three deciding factors, namely depth, position and orientation of a neural source, and the resulting scalp potentials. Second, we quantify the expected loss in signal amplitude for three ear-EEGs that are compared to whole-head 128-channel EEG: the cEEGrid, a horizontally oriented bipolar channel and a vertically oriented bipolar channel. Additionally, it is assumed that the sensitivity of the cEEGrid (i.e. the highest signal amplitude) relies on a mixture of all channels. An alternative hypothesis is that only a few channels reflect the highest amplitude for the majority of sources. If the goal would be to record with as few channels as possible, these cEEGrid electrodes would be the ones that could be discarded. Third, we compute a fine-grained sensitivity map of both the cEEGrid and cap-EEG for capturing dipole sources distributed over the entire cortex. Finally, we discuss the implications of our findings and will provide recommendations for the design of ear-EEG solutions.

## Methods

With forward modeling, we illustrate some basic principles that play a role for the development of ear-EEG systems. All simulations presented here were conducted with the freely available Brainstorm toolbox (Tadel et al. (2011)) for MATLAB.

### EEG Channels

**Fig. 1:**
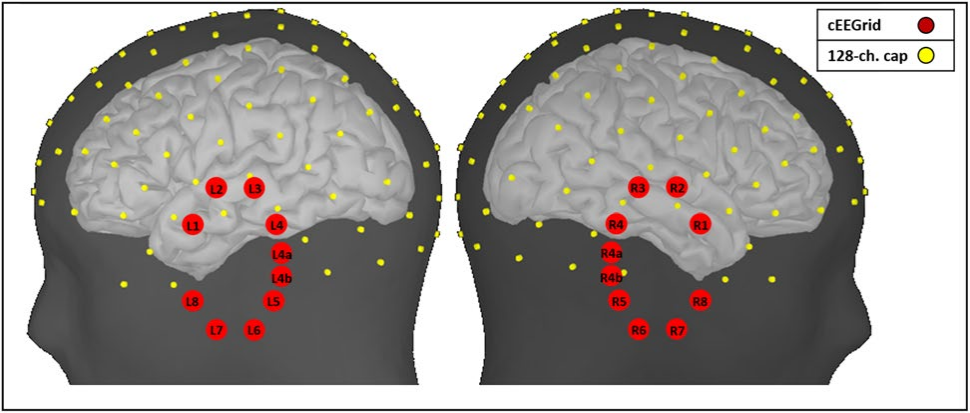
Electrode positions of cEEGrid electrodes (red) and the 128-channel cap electrodes (yellow)

For the simulations, different electrode setups as shown in Fig. 1 were used: a 128-channel EEG-cap (international 10–5 system), the cEEGrid around the left ear and one simulation with two cEEGrids around the left and right ear. Additionally, two bipolar electrodes with positions taken from the left cEEGrid were chosen (L1 and L4, with a horizontal orientation, L3 and L6, with a vertical orientation). Throughout all simulations, for all channels used for analysis, the amplitude between pairs of electrodes was computed. The cEEGrid electrode locations were transferred into the Brainstorm coordinate-system (maintaining their respective fixed inter-electrode distances) and their positions were then projected onto the scalp of the default head (Colin 27, Holmes et al. (1998)) in Brainstorm. For the same head, the 128-channel cap template provided by Brainstorm (BrainProducts, EasyCap 128) was used.

### Anatomy

Brainstorm provides a solution for the forward modeling of cortical activation (Mosher et al. (1999)). For the simulations in this paper a four-shell anatomical image of the head (Colin 27) with segmentations for skin, skull, CSF and brain was used. The head model was computed using the Boundary Element Method (BEM, Gramfort et al. (2010)). The default Brainstorm conductivity values for the head are given as ratios between tissue types: 0.33 S/m (brain/CSF), 0.0042 S/m (CSF/skull), and 0.33 S/m (skull/skin). In contrast to Kappel et al. (2019), this model does not include a detailed description of the anatomy of the ear (concha or ear-canal). Since most anatomical scans used for source localization in ear-EEG do not include additional modeling of the ear-canal, it was decided to use the standard T1-weighted anatomical image provided by Brainstorm. Moreover, while this extension of imaging the ear-canal in a more detailed manner adds to precision, the emerging changes for the forward model can be expected to be less impactful for electrodes placed around the ear compared to those placed directly on the ear-canal or the concha.

### Source Space for the Brain Volume and the Cortex Surface

To demonstrate the effects of various source properties on the recorded signal, sources were placed in the brain volume, as this allowed for flexible positioning of the sources along several dimensions, such as depth and orientation of the source relative to a sensor. The source grid consisted of 12,891 isotropic points with a grid resolution of 5 mm within the brain volume. For this model with unconstrained sources, Brainstorm provides three orthogonal orientations per dipole following the x-, y- and z-axis. This results in a 12,891 * 3 = 38,673 matrix for the specification of all dipoles. For this volume-based head model, all dipole properties used for simulations of dipole orientation, position and depth are specified in Sect. Source orientation, Source position, Source depth.

For the simulation of sensitivity of ear-EEG compared to cap-EEG, the source space was restricted to the grey matter. The orientation of the dipoles was restricted to be perpendicular (normal) to the cortex surface (i.e. the default in the brainstorm toolbox). This results in 15,002 source points following the shape of the grey matter surface with constrained orientations.

### The Influence of Source Properties on the Recorded Signal

As mentioned in Sect. Introduction, the signal that is recorded with electrodes on the scalp is dependent on the individual characteristic of a person’s head. That includes for example the individual head geometry, conductivities of different tissue types and properties of active cortical tissue. Importantly for use here, how the activity in a given functional anatomical structure is reflected on the scalp depends on the orientation of that area relative to the recording electrodes and is hence dependent on the unique cortical folding of a person. Specifically, the signal amplitude as recorded by any pair of EEG electrodes depends on the orientation (i.e. the direction of the positive and negative part of the dipole), the position (e.g. above/behind an electrode) and the depth of the source (i.e. the distance between recording electrode and source). The term “depth” in this context should be understood as the distance between cEEGrid electrodes and the current dipole. Likewise, the term “position” refers to the dipole position relative to the cEEGrid. Only the orientations of the simulated dipoles are given independently from the cEEGrid (e.g. “anteriorly”, to indicate the dipole orientation following the course of the x-axis of the coordinate system).

To illustrate the dependency of these source properties on the measured signal, single dipoles were modeled in the brain volume and each factor was varied systematically. Throughout this study and for all simulations, the amplitude of all sources was always set to 1 *nA/m* and there was always only one dipole or dipole patch active at any time. For the simulated signal, an average reference was used. In addition, all simulations in this study were carried out noise-free, since realistic estimates of source strengths relative to noise would have required a much more costly simulation, including knowledge about the exact nature of the source in terms of strength and location.

### A Multi-Electrode Ear-EEG

Scalp-EEG is sensitive to the synchronized activity of large populations of neurons. Because neural activity is not always confined to very small areas of cortex, we modeled here sources arising from larger areas (patches) whose spatial extends follow the pattern of gyri and sulci of the folded cortex and are therefore more suitable for displaying the sensor-source relationship. We defined the left hemisphere as a region of interest for the sensitivity of the left cEEGrid and its bipolar subsets of channels. Due to its distance to the electrodes, we decided to leave out the medial part of the hemisphere that is facing the interhemispheric fissure. While the specification of the anatomical boundaries may be regarded as incomplete, publication of all scripts, including the scout defining the parcellation of the cortex surface (see Code availability) along with the manuscript will allow readers to exactly reproduce our boundary definitions. This area was parceled into a total of 50 patches (with a mean size of 14.2 cm^2^ and a standard deviation of 3.1 cm^2^). Due to the unequal spacing of the vertices on the cortex mesh, the regions derived from parcellation differ slightly in size.

The sensitivity of high-density cap-EEG, cEEGrid and the bipolar channels was computed for each patch individually. For this, activity was seeded in all vertices forming a patch. Forward modeling then computed the respective lead field potentials at each electrode for each patch, linking the sources to the differences in potential measured at every sensor location. For each cap and cEEGrid, the channel that captured the activity of that patch best (i.e. recorded the maximal amplitude) was identified, respectively. This means, for each patch, every combination of electrode pairs (190 possible combinations for the 20 cEEGrid electrodes and 8128 for the 128 cap-EEG electrodes) was compared to find the one reflecting the highest amplitude per setup. As cap-EEG is the conventional way to measure EEG, we are showing the relative signal loss of the ear-EEG systems compared to a full cap-EEG. The sensitivity of the ear-EEG systems for a given area is expressed as the percentage of signal change relative to the cap-EEG channels with the highest amplitudes. If the amplitude as measured with cEEGrid electrodes is smaller than the amplitude measured with cap electrodes we speak of *signal loss*, if the cEEGrid amplitudes are larger than the cap amplitudes we speak of *signal gain*.

### A Whole-Brain Sensitivity Map

For an overall estimate of the capacity of bilaterally placed cEEGrids, we calculated a sensitivity map for the entire cortex. For this, the activity of all sources on the cortex surface was set to zero, except for one active dipole with an amplitude of 1 *nA/m*. Thereby, activity was always only simulated for a single dipole at a time, forward modeling the signal to simulate potentials at channel level. Independently for the cap and the cEEGrid electrodes, the respective channel that captured the activity of that dipole best (i.e. recorded the maximal amplitude) was identified as described in the section above. For visualisation, the resulting amplitude value was then mapped onto the cortex surface at the position of the seed. The procedure was repeated for all 15,002 source points on the cortex surface, resulting in one sensitivity map for the cap and one sensitivity map for the cEEGrid. Both maps can be found in the Supplementary information of the online version of this article. A link to the code used to compute all simulations, as well as illustrating short videos of the source properties orientation, position and depth can be found in the section Code availability.

## Results

**Fig. 2:**
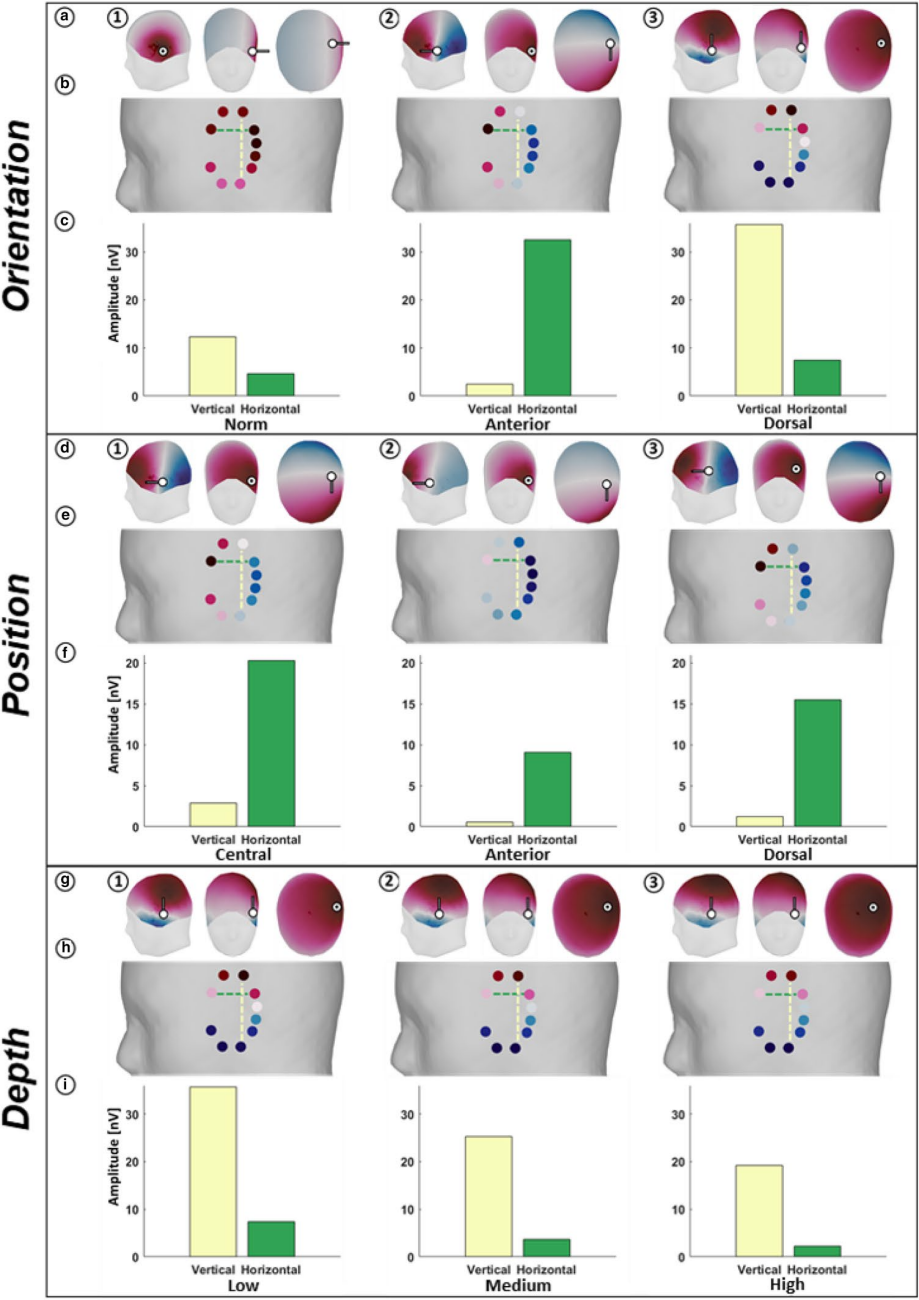
Illustration of the sensor-source relationship, dependent on the orientation (top panel), position (middle panel) and depth of a source (bottom panel). In the top panel (Orientation) the position of the modeled dipole is fixed, and the orientation is varied: the dipole has a lateral (first column), anterior (second column) or dorsal orientation (third column). In the middle panel (Position) the orientation of the modeled dipole is fixed (anterior), and the position is varied. The dipole is located closest to the geometric center of the cEEGrid (first column), shifted 4.5 cm in an anterior direction (second column), or shifted 4.5 cm in a posterior direction (third column). In the bottom panel (Depth) only the depth of a dipole, with dorsal orientation, is varied relative to the cEEGrid. The dipole is located in the grey matter (first column), shifted 1.5 cm in the medial direction (second column), or shifted 3 cm in the medial direction (third column). This movement denotes the movement of the dipole into the brain volume, not the distance of each individual electrode to the source. In the top row of each panel (**a**, **d**, **g**) the sagittal, axial and coronal views of the head are shown, with the electric potentials represented as a topographic map on the surface of the scalp. The white dot indicates the position of the dipole, the black line its direction. In the middle row of each panel (**b**, **e**, **h**) the electric potential at individual cEEGrid electrodes is shown. The yellow and green dotted lines indicate one vertical and one horizontal bipolar channel formed by the respective electrodes. The bottom row of each panel (**c**, **f**, **i**) shows the absolute amplitude in *nV* measured by these bipolar channels. Note that the limits of the y-axis of F are different from **c** and **i**

Several simulations were conducted to show how different features of a current dipole source influence the resulting topographies and therefore the measured signals. To illustrate the orientation, position and depth of each dipole, these are represented as a vector originating from the source point with the positive part of the dipole following the direction of the line in Fig. 2adg. For each topography, the distributions of scalp potentials for the modeled dipole from a sagittal, a coronal and an axial perspective are displayed. Below in BEH, the signal amplitudes as measured by the cEEGrid channels are shown on the scalp and refer to the plots in ADG. To compare different electrode arrangements, two bipolar channels were chosen, one with a vertical and one with a horizontal orientation, as displayed in BEH. The resulting signal strength for each channel is shown in Fig. 2cfi.

### Source Orientation

To demonstrate the influence of the orientation of a source, a dipole was placed on the grid point closest to the center of the left cEEGrid, in the anterior part of the inferior temporal gyrus. This way, all cEEGrid electrodes had similar distances to the source. Three vectors representing the orientations of one active dipole were set: The normal vector to the hypothetical plane put up by the positions of the cEEGrid electrodes (a vector pointing directly onto the cEEGrid) and the two orthogonal vectors to the first one, pointing in a dorsal and anterior direction (Fig. 2a1, 3). In the orientation panel of Fig. 2a1, the positive part of the dipole potential covers the entire cEEGrid. For any bipolar channel one would measure amplitudes close to zero, because all electrodes are located over the positive part of the dipole. In Fig. 2a2, 3, the orientation of the dipole changes. The isopotential line of the dipole, i.e. the area where positive changes into negative, runs exactly through the vertical and horizontal center line of the cEEGrid, respectively. In this case there are electrodes on the negative as well as on the positive dipole part. Depending on the orientation of the dipole, it is either a horizontally oriented electrode pair (Fig. 2a2) or a vertically oriented electrode pair (Fig. 2a3) that optimally captures the signal of interest (i.e. the largest amplitude), while the other electrode pair measures close-to-zero amplitudes.

**Fig. 3:**
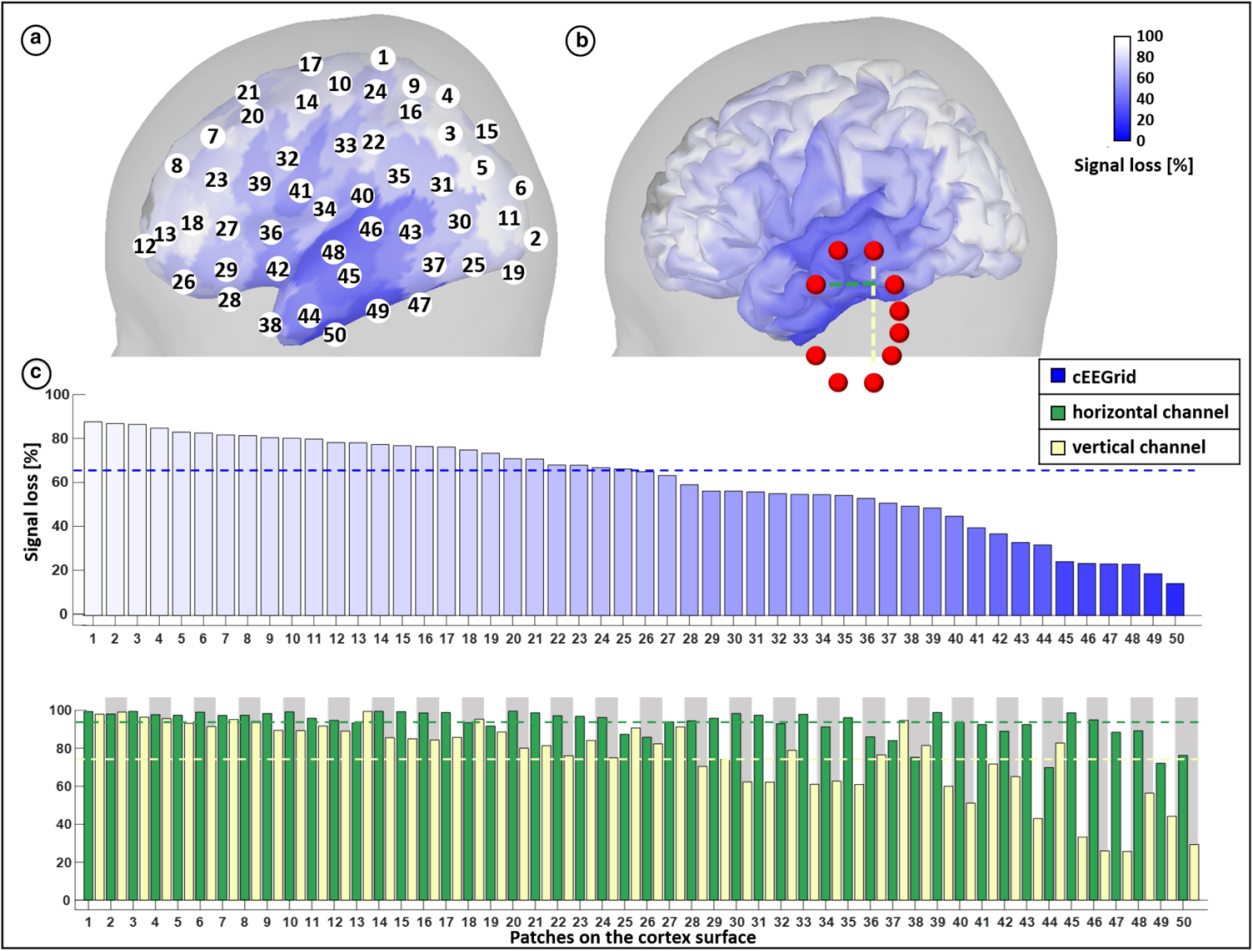
Comparison of 128-channel and ear-EEG sensitivity. The percentage loss in amplitude measured from the cEEGrid compared to cap-EEG is shown. **a** Percentage signal loss of ear-EEG relative to cap-EEG is shown for 50 regions (patches). The patches are color-coded and numbered (1–50) based on the signal loss for the cEEGrid relative to the cap (100–0%), from white (highest signal loss) to blue (lowest signal loss). **b** Shows the same patches on an uninflated cortex with the locations of the cEEGrid electrodes (red). The two dotted lines in B represent the horizontally oriented channel (green) and a vertically oriented channel (yellow). **c** The upper bar chart shows the percentage signal loss of the cEEGrid, ordered from high to low. The numbers on the x-axis correspond to the numbers in A. The lower bar chart shows the percentage signal loss of the bipolar channels (see b), the order was inherited from the upper bar chart. The signal loss of the horizontal channel is shown in green, the signal loss of the vertical channel is shown in yellow. The bars are grouped per patch by an alternating grey and white background for easier assignment. The dotted lines in both bar charts represent the average signal loss over all patches

### Source Position

To demonstrate the influence of the position of a source relative to the electrodes, a source was set into the brain volume, located on the imaginary normal vector from the center of the cEEGrid into the brain. From this first dipole, two additional dipoles were placed, one in an anterior direction, the other in the dorsal direction, both with a distance of 4.5 cm to the first dipole. Therefore, these two dipoles (as displayed in Fig. 2d2 and d3) both have the same distance to the center of the cEEGrid. This way, both the distance and the orientation of the two dipoles relative to the cEEGrid-center stay the same, only the positions of the dipoles have changed. The first dipole (with anterior orientation) was best captured by a horizontally oriented electrode pair, as explained in the section above (Fig. 2d1). A shift of this dipole, either anteriorly or dorsally (Fig. 2d2, 3) led to a considerable decrease in the resulting amplitudes. Notably, the overall ratio of recorded amplitude strength between electrodes stayed the same, only the maximum amplitude that can be measured changed. In other words, the electrode pair measuring the highest amplitude would not change for all three positions, only the signal strength they receive. As can be seen in Fig. 2e, the appearance of the topographies was not constant over positions. This change was determined by the position of the source, because in Fig. 2e2, the isopotential line of the dipole runs outside of the “field of view” of the cEEGrid, i.e. almost all electrodes are on the negative part of the dipole while in Fig. 2e3 the isopotential line runs through the same positions as in Fig. 2e1.

### Source Depth

To demonstrate the influence of depth, the dipole closest to the center of the cEEGrid was used again as shown in Fig. 2g1. Going in the medial direction and deeper into the brain volume, two additional dipoles were placed at a distance of 1.5 cm and 3 cm (G2 and G3, respectively) to the first one. Note, that the values of 1.5 cm and 3 cm denote the movement of the dipole into the brain volume, not the distance between individual electrodes and the source. Because the electrodes of the cEEGrid are not in a perfect plane, both the distance of each channel and its orientation relative to the source slightly differ. The mean distance between electrodes and sources is 4.03 cm, 5.19 cm and 6.50 cm, respectively for each dipole. The sources had the same dorsal orientation as in Fig. 2a3, with the respective resulting topographies and amplitudes illustrated in HI 1–3. The decline of signal strength as a function of distance has been investigated most recently by Kappel et al. (2019). With this simulation, we want to replicate and extend these findings to around-the-ear electrodes. Therefore, in the depth panel in Fig. 2, the effect of a source moving away from the electrodes is illustrated. A source with fixed position and orientation was seeded and its distance to the electrodes was increased. The result can be seen in Fig. 2h and i: For the bipolar channels (horizontal and vertical), the signal amplitude drops with increasing depth.

### Comparison of Large-Scale Sensitivity

Figure 3a shows the simulated signal loss of a cEEGrid recording relative to a full cap recording for all patches. Temporal regions close to the cEEGrid show the lowest signal loss compared to high-density EEG, as can be seen more directly in Fig. 3b. The highest signal loss is observed for regions that are far away from the cEEGrid electrodes. On average, across all patches a 66.82% (*SD* 22.79%) signal loss of the cEEGrid compared to the full cap is observed. The minimum loss for the cEEGrid is 15.93% for temporal areas. The largest loss (96.70%) is observed for far away patches in the frontal and occipital areas. For the horizontal bipolar channel in C, there is an average signal loss of 93.38% (*SD* 7.23%, min 69.81%, max 99.53%). The vertical bipolar channel has on average a signal loss of 75.61% (*SD* 20.13%, min 25.71%, max 99.42%) compared to the cap-EEG. Between the two bipolar channels, the maximum difference in signal loss is 68.98% (patch 46).

**Fig. 4:**
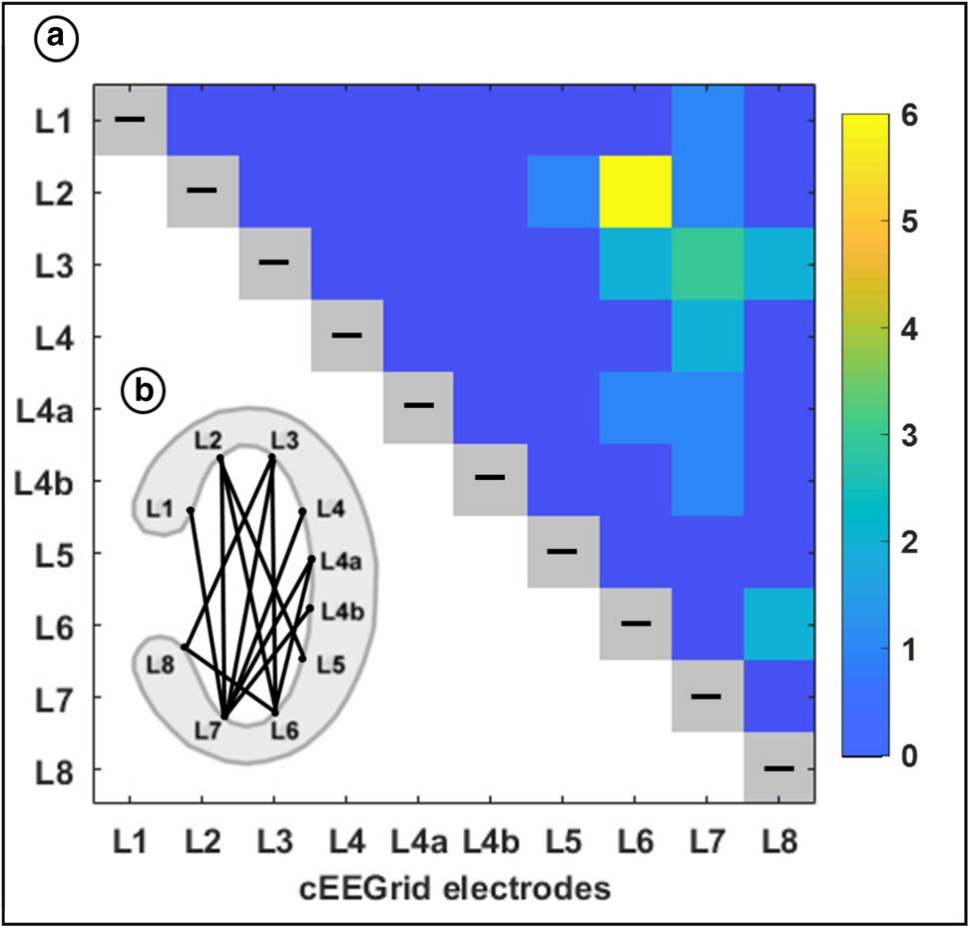
Illustration of the prevalence of bipolar cEEGrid channels measuring the highest amplitude from the source patches shown in Fig. 3. Additionally, the respective orientation of these high-performing channels is shown. **a** The grid shows all electrodes of the left cEEGrid on the x- and y-axis. The coloured squares indicate the number of times a channel measured the highest amplitude from a source. Values of zero (dark blue) for an electrode combination indicate that this channel (e.g. L1 and L2) never resulted in the highest amplitude measured from a source. L2 and L6, for instance, measured six times (yellow) the higher amplitude compared to all other channel combinations for six different sources. For this graph, only the patches with a signal loss below average (*n* = 23) were considered. **b** Shows the left cEEGrid with its electrode locations. A solid black line connects every electrode pair that measured the highest amplitude at least once

To understand what combinations of electrodes contribute the most to detecting a signal of interest (by measuring the highest amplitude), Fig. 4a shows the number of times each combination of electrodes recorded the highest amplitude from a patch and Fig. 4b shows the occurring connections on the cEEGrid (i.e. L1 and L2 never record the highest absolute amplitudes compared to the others, but L2 and L6 do it for six different sources). Here, we include only those patches that have a signal that is less than the average of 66.82% (*n* = 23). For patches with a very high signal loss, it is not informative which electrode pair captures the source best, because in this case, amplitudes recorded from all bipolar channels are close to zero. Their relative difference becomes meaningless and uninterpretable. First and foremost, it can be seen that each electrode was used at least once. From B, it becomes apparent that there were almost no horizontal channels recording the highest amplitudes for any source. In fact, for 52.17% (12 from 23) of the patches, the pairings of L2 with L6/L7, and L3 with L6/L7 recorded the highest amplitude.

### Comparison of Single-Dipole Sensitivity

**Fig. 5:**
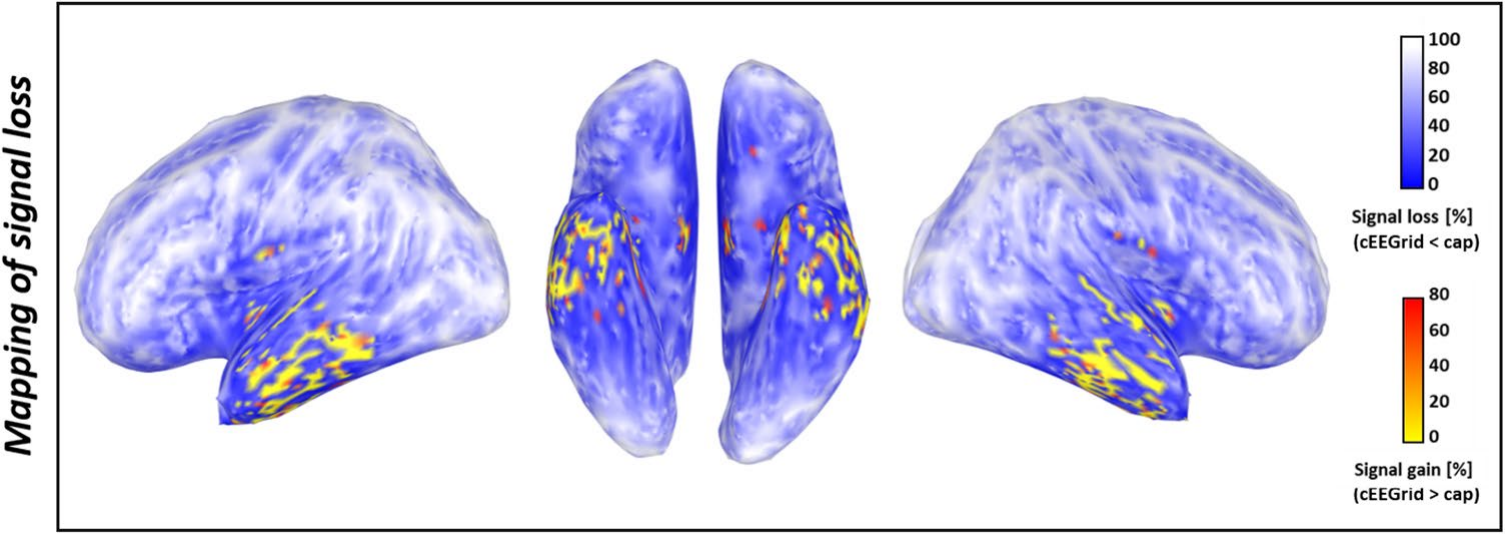
Illustration of the different sensitivities of the cEEGrid and 128-channel EEG to 15,002 single dipole sources distributed over the cortex surface. The colors on the cortex surface indicate the percentage difference in amplitude measured by the cEEGrid relative to cap-EEG for every dipole. In the left and right sagittal and the ventral view of the brain, this signal loss is color-coded from 100% (white) to 0% (dark blue). Areas colored from yellow to red indicate dipoles from which a cEEGrid channel measured a higher amplitude than any combination of cap electrodes (signal gain), where yellow indicates low signal gain and red indicates high signal gain

The parcelling of the cortex surface into patches in the section above gave a good idea about how some channels are more or less sensitive to different sources. To extend this approach and attain a whole-brain map of the sensitivity of the bilateral cEEGrids compared to the full cap, the sensitivity values of the cEEGrid were divided by the sensitivity values of the cap for each of the 15,002 sources on the cortex surface. Figure 5 therefore shows the percentage difference (signal loss/signal gain) in amplitude between the cap-EEG and the cEEGrids. It can be seen that areas with low signal loss are not confined to the temporal lobe alone. In total, for 296 of the 15,002 vertices (1.97%), there even was a signal gain (yellow–red areas), indicating that for these sources some cEEGrid channels recorded higher amplitudes than any cap channels. For the values with signal gain, an average of 8.50% was observed (*SD* 10.51%, min 0.01%, max 77.85%).

## Discussion

EEG beyond the classical laboratory environment has the potential to change our understanding of how the brain works by providing insights into neuronal processes in everyday life. Long term EEG recordings in everyday life, however, will remain limited for practical considerations in the number of electrodes that can be placed. Ear-EEG may provide a viable middle ground between transparency (Bleichner et al. (2015); Bleichner and Debener (2017)) and signal sensitivity. To make optimal usage of the small area in- and around the ear, and the relatively small number of electrodes that can be placed here, we need a better understanding of sensor-source relationships. Simulations as the ones provided in this study aim to elucidate this relationship. This will help developing better sensor technology, identifying possible ear-EEG applications and understanding inter-individual differences in ear-EEG recordings.

### Source Properties

We addressed the sensor-source relationship in EEG by showing how the amplitudes that can be recorded around the ear are dependent on the orientation, position and depth of the source of interest relative to the recording electrodes. These factors influence, first, whether it can be expected to reliably capture signals from a cortical location with ear-electrodes at all, and, second, which ear-electrodes are most sensitive for a given source. In terms of source orientation and position, our results demonstrate that while one electrode configuration may be highly sensitive to one source, it may be insensitive to another (see Fig. 2a1–3). While this point is trivial for experienced EEG researchers, it is an important factor when working with ear-EEG and a limited number of electrodes in and around the ears. From simulations in the depth panel in Fig. 2, it becomes apparent that deeper sources are harder to detect. As discussed in Kappel et al. (2019), the proximity of the source to the recording electrodes is one of several factors determining signal amplitude. That is, in an EEG recording, even with ideal orientation and position of a source, its signal still may not be observable at sensor level, due to the source’s distance to the electrodes and the resulting low signal amplitudes. Another influence that follows the same principle as the depth of the source and its distance to the electrodes is the signal strength. Even a source close to a channel might not be detectable if the electric potential difference is not strong enough (i.e. because of a too small population of neurons firing synchronously). However, a strong neural source at a large depth could still emit a signal with an amplitude high enough to be detected.

In summary, it should be recognized that orientation, position and depth are not fully independent properties of a source, but instead their complex combination determines what amplitudes can be recorded. These considerations are what should drive the placement of electrodes. While it is important to use a sufficiently high number of electrodes that are arranged in a way that captures as many different source orientations as possible, high user comfort, low visibility demands and technical constrains set some limitations.

### Comparison of Ear- and Cap-EEG

We quantified the expected loss in signal amplitude for three forms of ear-EEG to compare their capability in recording from different cortical areas. For the cEEGrid, our simulations of the signal loss relative to cap-EEG show that temporal areas exhibit the lowest signal loss, both for the patches in Fig. 3 and the more detailed mapping of signal loss in Fig. 5. This is an indication that one can measure neural activities from these areas with a quality comparable to classical EEG. For a direct comparison between ear-EEG results and cap-EEG results (e.g. as reported in Bleichner et al. (2016)), only a small difference in amplitude can be expected for these areas. This is likely due the cEEGrid covering space below as well as above the ear with a relatively high density of electrodes. These findings are reassuring for ear-EEG targeting auditory processes located within the temporal lobe, like the auditory cortex (Woldorff et al. (1993); Grady et al. (1997); Hine and Debener (2007)). However, for areas further away, one can expect lower amplitudes in comparison to what is described in the literature for cap-EEG. In this context, one should not pay too much attention to the exact ranking of the patches in Fig. 3a and b in terms of signal loss, as this measure is dependent on the random parcelling of the cortex. This indication was confirmed by the fine-grained mapping of signal loss in Fig. 5. It can be seen that in the left temporal lobe, for two adjacent areas, the cEEGrid showed very pronounced differences in sensitivity. In fact, the region with the cEEGrid’s highest sensitivity on the cortex was within high proximity to areas with signal losses of over 50%. These variations in the fine-grained map are due to differently oriented sources, which underlines that the sensitivity of ear-EEG (or cap-EEG) is not only a matter of distance but an interplay of several factors. This observation highlights the necessity of individualized head models for any kind of source modeling.

For Fig. 5, note that the signal losses for medial areas must be interpreted with caution: here, the signal losses for the cEEGrid were low on large parts of the brain, indicating that these sources can be recorded well. Yet, the ventral views of both the sensitivity map of the high-density EEG and the cEEGrid (see Supplementary Information for the sensitivity maps) show that low amplitudes were recorded from these regions for both devices. Therefore, low signal losses here do not indicate a high performance of the cEEGrid, but only that neither cap- nor ear-EEG captured signals from medial areas very well, i.e. the cEEGrid is equally unsuitable as the cap. Besides the medial sources, another aspect in Fig. 6 (see Supplementary Information) must be mentioned. As can be seen in the lateral views of the cortex, there is a peak sensitivity in the left temporal lobe for both the cap-EEG and the cEEGrid that is not present for the contralateral hemisphere. The authors attribute this difference to an anatomical characteristic that can be seen in the ventral view (middle column) of the specific brain used here: in the left temporal region (here displayed on the right side) there is a bulge that is not present on the right side. We assume that the increased proximity of this cortical patch to the electrodes is the reason for this one-sided peak. This observation shows exemplary that the exact results of our simulation approach are not to be taken as generalizable to the entire population. On the contrary, while more global patterns can be derived from this detailed sensitivity map, it highlights that individual anatomical differences will influence what can or cannot be recorded with ear-EEG.

### Arrangement of Ear-Electrodes

Manipulating the arrangement of electrodes leads to different amplitude recordings for various sources, in particular when done within a small area. Comparing the three different ear-EEGs, our results indicate that the bipolar channels have a higher signal loss relative to cap-EEG. Interestingly, while the patches with higher amplitude loss (being the ones far away from the electrodes that all uniformly measure amplitudes near zero) were comparable for both bipolar channel orientations, in the quarter with the lowest signal loss for the cEEGrid (areas 40–50), there were highly different signal losses (up to 68.98% difference in patch 46) for the two bipolar channels: in accordance with the results from Sect. Source orientation, when one orientation of a channel is suited well to capture a high amplitude from a source-patch, the orthogonal pair is most likely not. In the most extreme case, a cortical source that is captured well by one pair of electrodes will be “invisible” to another. For practical considerations, whether a single bipolar channel suffices to capture the signal of interest is difficult to answer a priory for an individual. Depending on the individual brain anatomy it may be sufficient for some people but not for others. The presence or absence of an (ERP) effect may hence be simply due to anatomical but not functional differences between people. A multi-channel setup will be less susceptible to this problem as the optimal channel configuration could be used for each participant and thereby reduce seemingly large inter-individual differences.

Besides the higher sensitivity to cortical sources when using several channels (instead of having only a single bipolar channel), multi-electrode setups are advantageous for pre-processing and analysis steps like artefact rejection and source localization. The quality of several algorithms depends highly on the spatial coverage and the number of electrodes used. Therefore, even if the optimal channel orientation and position for the detection of a signal was known a priori, a multi-channel EEG is still recommendable for high-quality measurements.

Our simulations show the limits of using only a single bipolar channel; a multi-channel setup will always provide a better sensitivity to a variety of neural sources. For some applications, however, a reduced number of electrodes may be wanted. It is therefore interesting to examine which electrodes could be discarded, if a minimal number of electrodes is of paramount importance. Therefore, in Fig. 4, the number of times a channel recorded the highest amplitude for a patch was counted. It can be seen that for the cEEGrid, no electrodes were redundant for optimally capturing differently oriented sources, since every electrode was at least once part of the channel that recorded the highest amplitude from a source. Yet, vertical channels (e.g. L2 and L7) are selected more frequently than horizontal ones (e.g. L8 and L5). The likely explanation is that vertically oriented electrode combinations have larger between-channel distances due to the ellipsoid shape of the cEEGrid. In general, a larger between-channel distance will lead to a higher amplitude, as discussed in Mirkovic et al. (2016), Bleichner et al. (2016) and Bleichner and Debener (2017).

### Implications for ear-EEG

In conclusion, the number and the C-shaped arrangement the cEEGrid electrodes allow to capture neural activity from a wide range of orientations. Nevertheless, it seems likely that the setup would benefit from a perfectly round shape to capture orientations more precisely. To summarize the implications of our findings and to provide recommendations for the design of ear-EEG solutions, there is a sufficient theoretical basis to measure ear-EEG from areas with low signal loss, namely in the temporal lobe and adjacent areas. Regarding the arrangement of the electrodes, a simulation in Bleichner and Debener (2017) hinted towards ear-EEG sensitivity being dependent on both source- and channel orientation. The present paper advances these findings with more distinct simulations of different source- and channel properties and demonstrates in a systematic way that a C-shaped multi-electrode setup will be sensitive to more cortical sources than a single, bipolar channel-setup and arguably allows to account better for inter-individual differences, which are known to exist in source orientations.

Using only a single electrode pair will therefore reduce the number of cortical sources that can be recorded. Of course, determining to which areas ear-EEG is sensitive to must be found in applied research, yet with simulating the sensitivity of an electrode arrangement to dipole sources, there is a clearer guideline for experimental decisions. Regarding the number of electrodes in the case of the cEEGrid, one has to weigh between a less obtrusive, unilateral and a more visible bilateral design that can record higher amplitudes. The cEEGrid in particular meets some of the favourable conditions addressed in Sects. Source orientation, Source position, Source depth, including sensitivity to different source orientations and high proximity to temporal sources, which is especially useful for measuring auditory evoked potentials. In this context, from previous research carried out with the cEEGrid, we already know of some effects that can be measured reliably, as stated in the introduction of this paper. Consequently, we expect a high chance of similar source amplitudes with low signal loss to be reliably found in a real-life setting. For research with a limited number of electrodes, our approach is made in a way that is generalizable to other forms of ear-EEG for additional simulations. This will help to relate existing EEG results obtained with cap-EEG to ear-EEG.

### Limitations

The simulation of cortical activity can produce useful models for EEG research. To clarify some of the basic structures that are important in this regard, we included several factors that are known to influence the recording of a signal into our simulations, such as precise head and brain geometry, different electrode setups and several different source properties. While revisiting some of the fundamental principles of EEG, we nevertheless aimed to keep our work as simple and illustrative as possible. Yet, our simulations can be extended to account for higher complexity: first, in our forward model we seed activity either in one vertex or in a group of neighbouring vertices. For the rest of the brain, no activity is assumed. Obviously, neural activity is never confined to only one part of the brain. So, despite the fact that our model indicates some degree of sensitivity for a given region, this activity may be masked by activity from other regions that is simply larger in magnitude. It may be the case that certain areas, despite having a good sensitivity in our simulation, are not recordable with the cEEGrid in reality. A solution for this may be to add noise to the model and see how robust the measurement of a given activity is against noise.

## Conclusion

Ear-EEG captures signals coming from various different brain areas. Our results provide an indication of how much signal loss can be expected for ear-EEG compared to conventional cap-EEG. For cortical activity from temporal areas, ear-EEG seems almost as suitable as cap-EEG and the measured amplitudes can be expected to be very similar. For areas further away, a larger reduction in the amplitude of signals of interest should be anticipated. In any case, our results clearly confirm the advantage of multiple electrodes arranged systematically around the ear.

## Electronic supplementary material

Below is the link to the electronic supplementary material.

Supplementary file1 (DOCX 739 kb)

## Data Availability

The code used to compute all simulations discussed in this study and illustrating short videos of dipole sources with varying orientation, position and depth (3Dorient.avi/3Dpos.avi/3Ddepth.avi) are available via 10.6084/m9.figshare.11907801.
