# Supplementary material for: The Sensitivity of Ear-EEG: Evaluating the Source-Sensor Relationship Using Forward Modeling
Source: Brain Topogr. 2020 Aug 24;33(6):665–76. doi: 10.1007/s10548-020-00793-2 (PMC7593286; doi:10.1007/s10548-020-00793-2)
Supplement: Supplementary file 1 — Supplementary file1 (DOCX 739 kb) [file 10548_2020_793_MOESM1_ESM.docx]

# Supplementary information


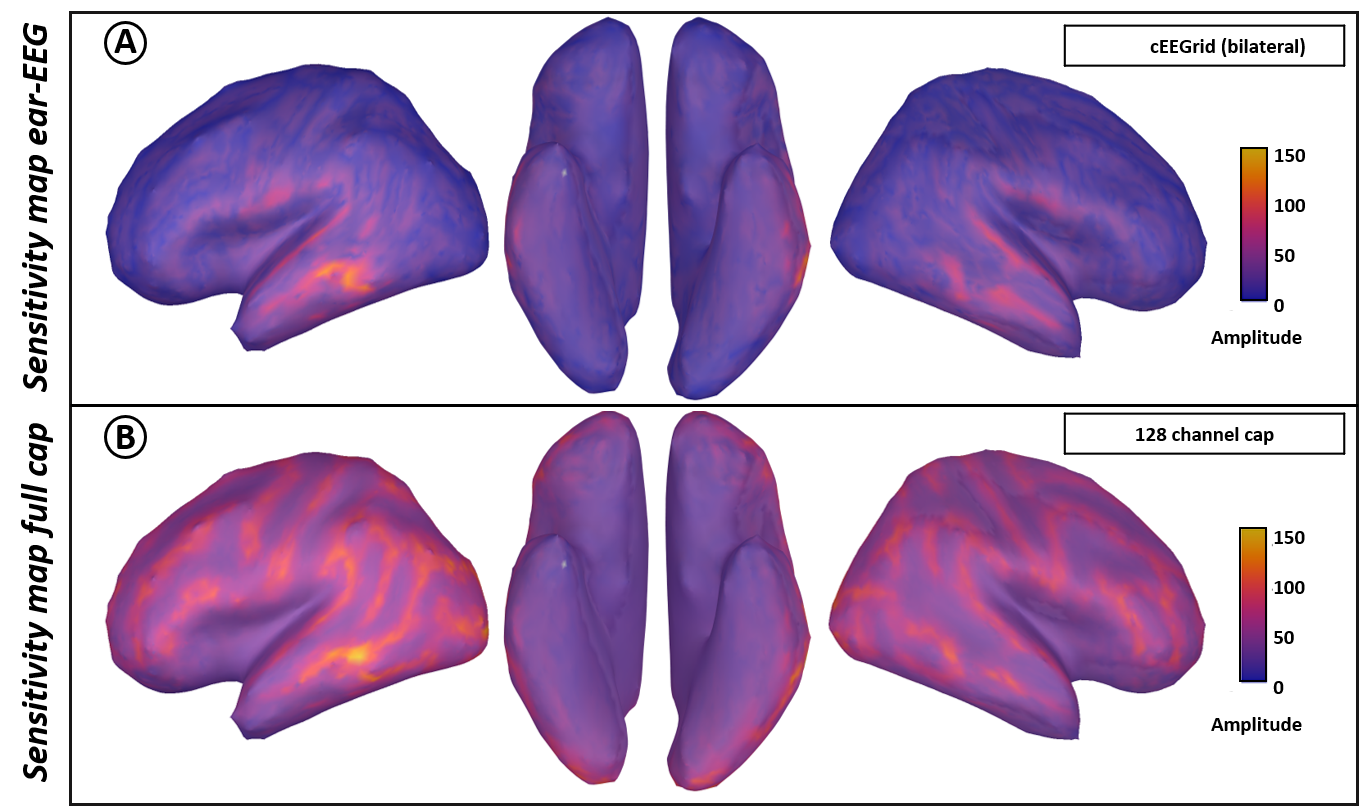


Figure 6: Sensitivity maps for the cEEGrid and cap-EEG. Shown are the left and right sagittal and the ventral view of the cortex surface. The maximum amplitude that each device can measure from each point on the cortex surface is shown. A) displays the sensitivity map for the bilateral cEEGrid, B) the sensitivity map for the 128-channel cap. Yellow represents higher amplitudes, purple lower ones.
